# Supplementary material for: Properties and Degradation Performances of Biodegradable Poly(lactic acid)/Poly(3-hydroxybutyrate) Blends and Keratin Composites
Source: Polymers (Basel). 2021 Aug 12;13(16):2693. doi: 10.3390/polym13162693 (PMC8399615; doi:10.3390/polym13162693)
Supplement: Supplementary file 1 [file polymers-13-02693-s001.zip › polymers-1322973-SI.pdf]

## Supporting Information

# Properties and Degradation Performances of Biodegradable Poly(lactic acid)/poly(3-hydroxybutyrate) Blends and Keratin Composites.

Danko M.[1,*], Mosnáčková K.[1], Vykydalová A.[1], Kleinová A.[1], Puškárová A.[2], Pangallo D.[2], Bujdoš, M.[3], Mosnáček J.[1,4].

*[1] Polymer Institute, Slovak Academy of Sciences, Dúbravská cesta 9, 845 41 Bratislava, Slovakia*
*[2]Institute of Molecular Biology, Slovak Academy of Sciences, Dúbravská cesta 21, 845 51 Bratislava, Slovakia;*
*[3] Institute of Laboratory Research on Geomaterials, Faculty of Natural Sciences, Comenius University in Bratislava, Mlynská dolina, 842 15 Bratislava, Slovakia;*
*[4] Centre for Advanced Materials Application, Slovak Academy of Sciences, Dúbravská cesta 9, 845 11 Bratislava, Slovakia;*

[*] *corresponding author: martin.danko@savba.sk*

Content:

1. **Figure S1**: Optical microscopy pictures of PLA/PHB/ATBC neat polymer matrix, keratin filler and PLA/PHB/ATBC/keratin composites with 1, 5, 10 and 20 wt% of keratin filler content at 150× magnification.

2. **Table S1**: Molecular characteristics of PLA/PHB, PLA/PHB/ATBC neat matrices and composites with varying keratin content from 1 to 20 wt%.

3. **Table S2**: Parameters obtained from DSC records of PLA/PHB and PLA/PHB/ATBC neat foils and keratin composites with varying keratin content from 1 to 20 wt% prepared by hot-pressing procedure.

4. **Figure S2**: DSC second heating run of PLA/PHB and PLA/PHB/ATBC neat foils and keratin composites with varying keratin content from 1 to 20 wt% prepared by hot-pressing procedure.

5. **Figure S3**: TGA and DTG curves of PLA, PHB and Keratin bare materials.

6. **Figure S4**: $^1$H NMR spectra of of PLA/PHB/ATBC/K-5 composite with 5 wt% of keratin in matrix before (upper) and after hydrolysis in 0.01 M NaOH (36 days, 78% extractable products) (down).

7. **Table S3**: Parameters obtained from DSC records of PLA/PHB/ATBC neat foil and keratin composites with varying keratin content from 5 to 20 wt% at the end of hydrolysis.

8. **Figure S5:** DSC second heating run of PLA/PHB/ATBC neat foil and keratin composites with varying keratin content from 5 to 20 wt% at the end of hydrolysis.

9. **Figure S6**: Average number molar masses of PLA/PHB/ATBC blend and its composites with varying keratin content from 1 to 20 wt% before and after the 1 month microbial test.

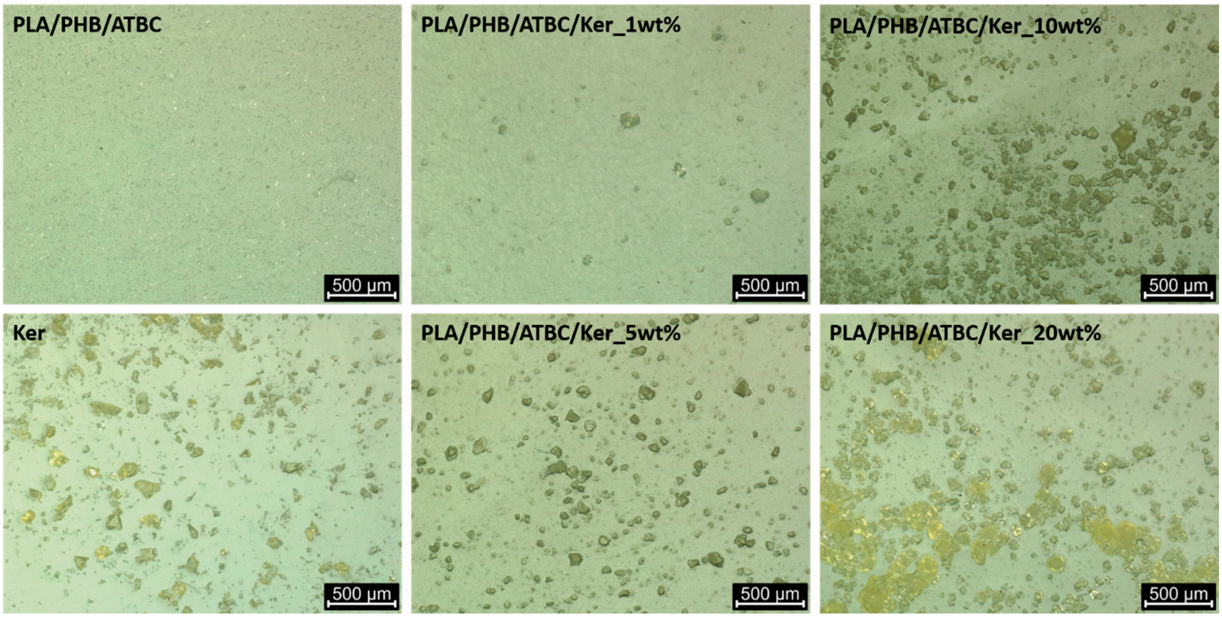


***Figure S1**: Optical microscopy pictures of PLA/PHB/ATBC neat polymer matrix, keratin filler and PLA/PHB/ATBC/keratin composites with 1, 5, 10 and 20 wt% of keratin filler content at 150× magnification.*

***Table S1**: Molecular characteristics of PLA/PHB, PLA/PHB/ATBC neat matrices and composites with varying keratin content from 1 to 20 wt%.*

| **Sample** | **Casted** | | **Compression-molded** | |
|---|---|---|---|---|
| | as prepared | treated 80°C, 16 h | as prepared | treated 80°C, 16 h |
| | $M_n$, $M_w$, Đ g mol$^{-1}$ | $M_n$, $M_w$, Đ g mol$^{-1}$ | $M_n$, $M_w$, Đ g mol$^{-1}$ | $M_n$, $M_w$, Đ g mol$^{-1}$ |
| **PLA/PHB** | 47 100, 100 500<br>2.13 | 51 200, 115 000<br>2.25 | 44 200, 93 800<br>2.11 | 43 700, 91 800<br>2.10 |
| **PLA/PHB/ATBC** | 45 700, 93 400<br>2.05 | 45 400, 93 100<br>2.05 | 43 200, 92 000<br>2.11 | 43 600, 90 100<br>2.07 |
| **PLA/PHB/ATBC/ K-1wt%** | 42 700, 89 600<br>2.10 | 45 400, 89 300<br>1.97 | 43 200, 83 000<br>1.91 | 42 500, 88 300<br>2.08 |
| **PLA/PHB/ATBC/ K-3wt%** | 43 700, 86 100<br>1.97 | 44 000, 89 400<br>2.03 | 37 300, 82 400<br>2.21 | 39 800, 90 100<br>2.26 |
| **PLA/PHB/ATBC/ K-5wt%** | 41 100, 88 200<br>2.15 | 38 500, 82 300<br>2.14 | 34 300, 82 800<br>2.41 | 38 200, 82 600<br>2.16 |
| **PLA/PHB/ATBC/ K-10wt%** | 36 100, 72 200<br>2.00 | 37 800, 80 300<br>2.12 | 33 200, 79 800<br>2.40 | 35 900, 83 000<br>2.31 |
| **PLA/PHB/ATBC/ K-20wt%** | 31 500, 66 000<br>2.09 | 32 900, 76 900<br>2.34 | 28 300, 64 200<br>2.27 | 27 000, 64 800<br>2.40 |

***Table S2**: Parameters obtained from DSC records of PLA/PHB and PLA/PHB/ATBC neat foils and keratin composites with varying keratin content from 1 to 20 wt% prepared by compression-molding procedure.*

| **Sample** | $T_g$ (°C) | $T_{cc}$ (°C) | $T_m$ (°C) | $\Delta H_{m1}$ (J g$^{-1}$) | $\Delta H_{m2}$ (J g$^{-1}$) | $\chi_c$ (%) |
|---|---|---|---|---|---|---|
| PLA/PHB | 51.3 | 121.5 | 149.1/170.7 | 36.25 | 33.46 | 35.88 |
| PLA/PHB/ATBC | 32.4 | 99.3 | 148.2/167.6 | 34.53 | 31.46 | 34.18 |
| PLA/PHB/ATBC/K-1 | 30.1 | 103.0 | 141.2/167.0 | 33.17 | 33.65 | 32.83 |
| PLA/PHB/ATBC/K-3 | 29.3 | 96.9 | 147.5/165.9 | 38.55 | 38.33 | 38.16 |
| PLA/PHB/ATBC/K-5 | 30.9 | 98.1 | 147.7/166.7 | 35.17 | 33.63 | 34.81 |
| PLA/PHB/ATBC/K-10 | 30.7 | 97.5 | 147.7/166.3 | 34.65 | 37.66 | 34.30 |
| PLA/PHB/ATBC/K-20 | 26.5 | 93.2 | 146.7/165.1 | 31.53 | 35.32 | 31.21 |

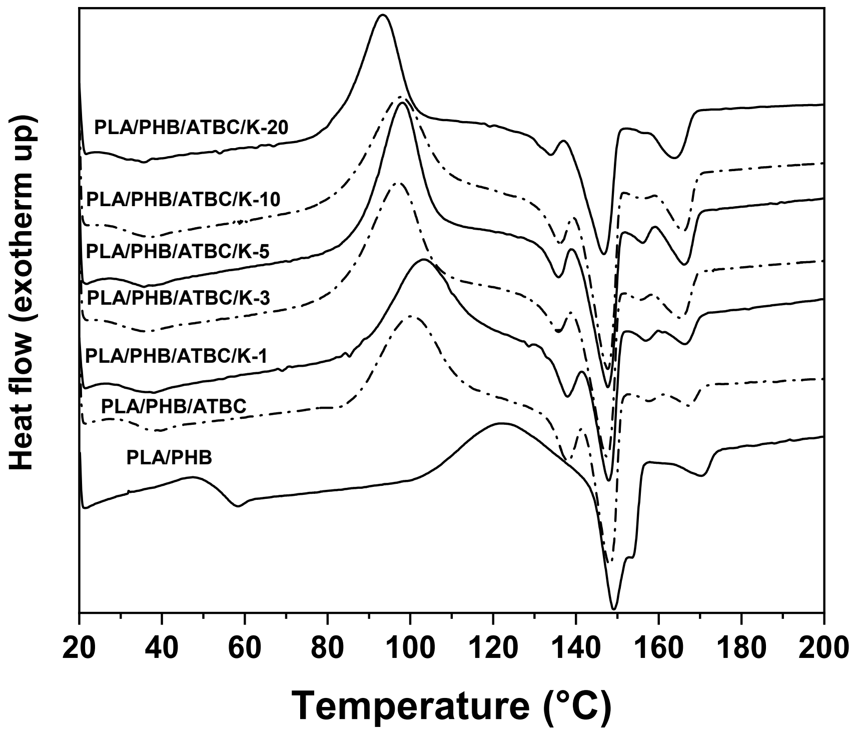


***Figure S2***: *DSC second heating run of PLA/PHB and PLA/PHB/ATBC neat foils and keratin composites with varying keratin content from 1 to 20 wt% prepared by compression-molding procedure.*

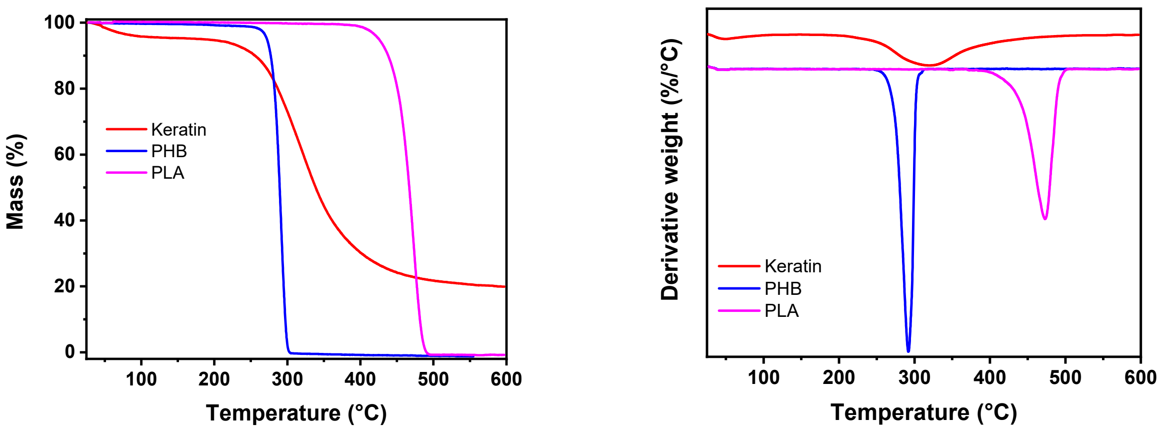


***Figure S3***: *TGA and DTG curves of PLA, PHB and keratin bare materials.*

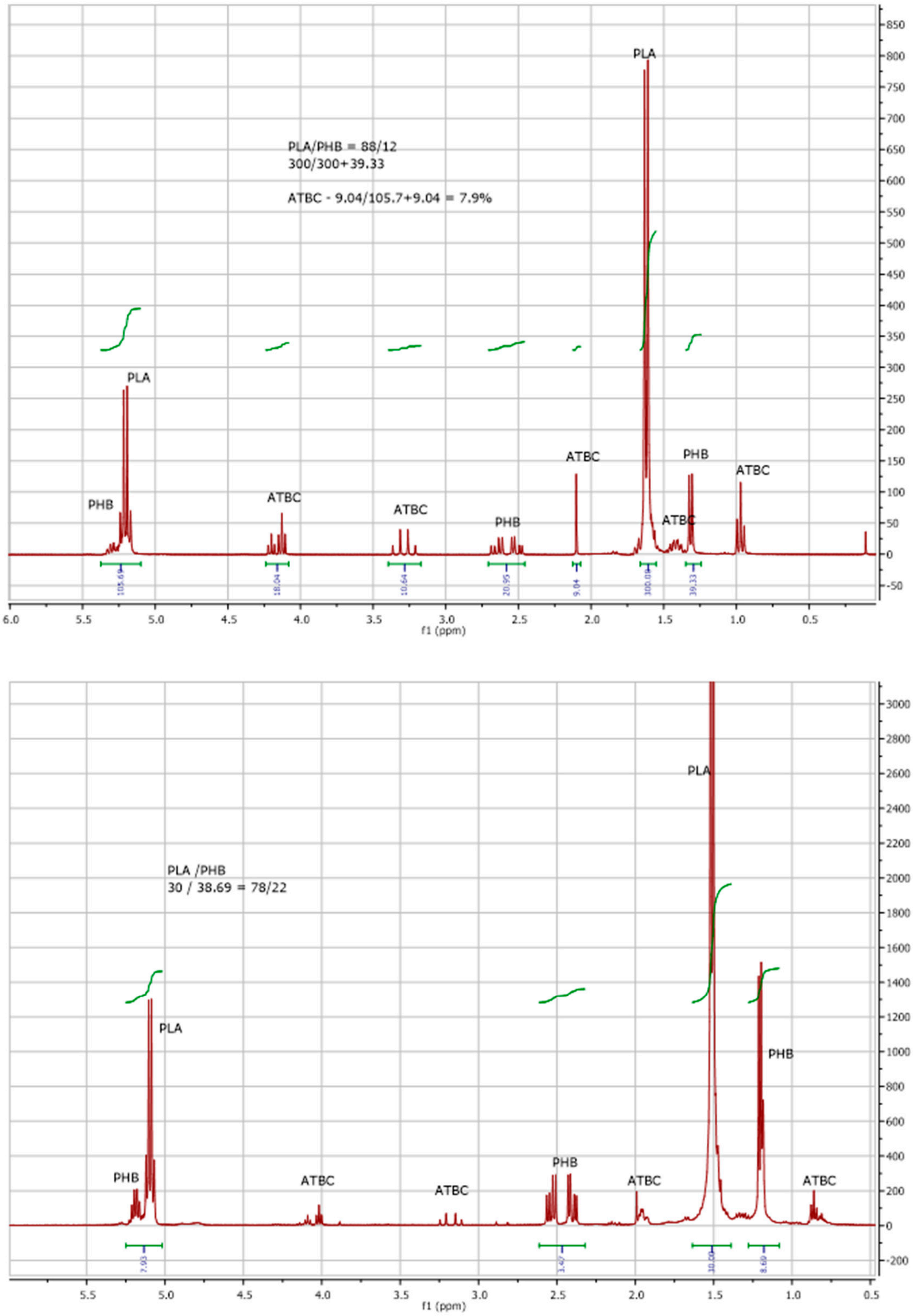


***Figure S4***: *$^{1}$H NMR spectra of of PLA/PHB/ATBC/K-5 composite with 5 wt% of keratin in matrix before (upper) and after hydrolysis in 0.01 M NaOH (36 days, 78% extractable products) (down).*

***Table S3****: Parameters obtained from DSC records of PLA/PHB/ATBC neat foil and keratin composites with varying keratin content from 5 to 20 wt% at the end of hydrolysis.*

| Sample | $T_g$ (°C) | $T_m$ (°C) | $\Delta H_{m1}$ (J g$^{-1}$) | $\chi_c$ (%) |
|---|---|---|---|---|
| PLA/PHB/ATBC | 54.9 | 149.6/164.3 | 34.53 | 49.31 |
| PLA/PHB/ATBC/K-5 | 52.6 | 150.1/165.6 | 49.82 | 50.11 |
| PLA/PHB/ATBC/K-10 | 52.5 | 150.2/163.5 | 50.63 | 47.63 |
| PLA/PHB/ATBC/K-20 | 51.6 | 148.9/161.6 | 48.12 | 36.80 |

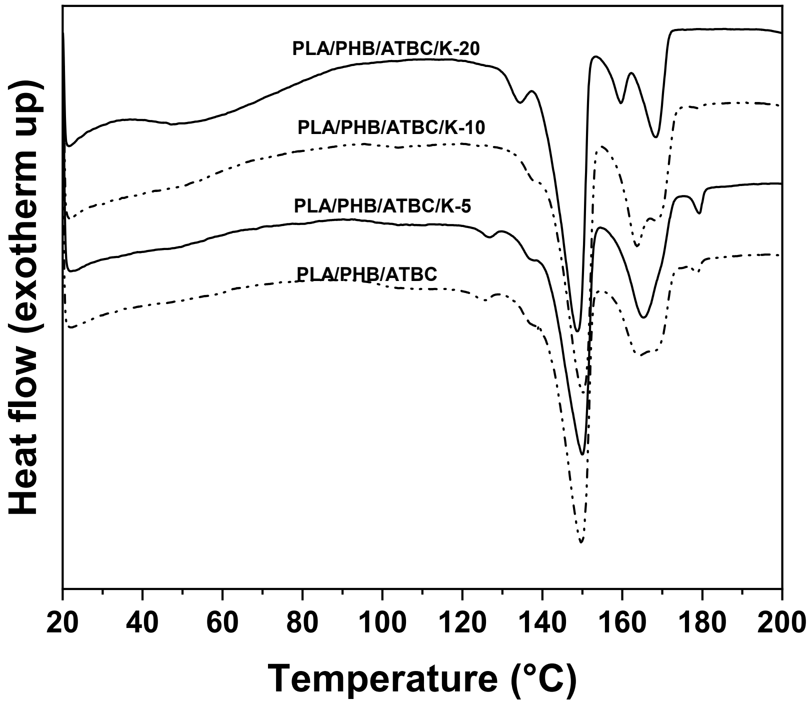


**Figure S5:** *DSC second heating run of PLA/PHB/ATBC neat foil and keratin composites with varying keratin content from 5 to 20 wt% at the end of hydrolysis.*

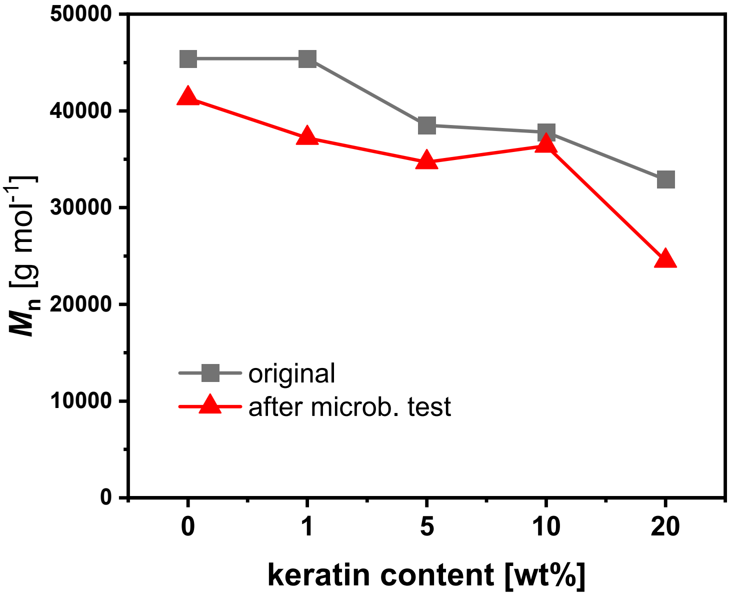


***Figure S6****: Average number molar masses of PLA/PHB/ATBC blend and its composites with varying keratin content from 1 to 20 wt% before and after the 1 month microbial test.*
